# An online tool for predicting ovarian reserve based on AMH level and age: A retrospective cohort study

**DOI:** 10.3389/fendo.2022.946123

**Published:** 2022-07-22

**Authors:** Yong Han, Huiyu Xu, Guoshuang Feng, Haiyan Wang, Kannan Alpadi, Lixue Chen, Mengqian Zhang, Rong Li

**Affiliations:** ^1^ Department of Thoracic Surgery, Zhejiang Provincial People’s Hospital, Affiliated People’s Hospital, Hangzhou Medical College, Hangzhou, China; ^2^ Key Laboratory of Tumor Molecular Diagnosis and Individualized Medicine of Zhejiang Province, Zhejiang, China; ^3^ Center for Reproductive Medicine, Department of Obstetrics and Gynecology, Peking University Third Hospital, Beijing, China; ^4^ Key Laboratory of Assisted Reproduction, Ministry of Education, Beijing, China; ^5^ National Clinical Research Center for Obstetrics and Gynecology, Beijing, China; ^6^ Beijing Key Laboratory of Reproductive Endocrinology and Assisted Reproductive Technology, Beijing, China; ^7^ Big Data Center, Beijing Children’s Hospital, Capital Medical University, National Center for Children’s Health, Beijing, China; ^8^ The Predict Health, Houston, TX, United States

**Keywords:** age, anti-müllerian hormone (AMH), online tool, ovarian reserve, predict

## Abstract

**Purpose:**

To establish a more convenient ovarian reserve model with anti-Müllerian hormone (AMH) level and age (the AA model), with blood samples taken at any time in the menstrual cycle.

**Methods:**

We have established this AA model for predicting ovarian reserve using the AMH level and age. The outcome variable was defined as poor ovarian response (POR) with <5 oocytes retrieved during assisted reproductive technology treatment cycles. Least Absolute Shrinkage and Selection Operator logistic regression with 5-fold cross validation methods was applied to construct the model, and that with the lowest scaled log-likelihood was selected as the final one.

**Results:**

The areas under the receiver operating characteristic curve for the training, inner, and external validation sets were 0.862, 0.843, and 0.854 respectively. The main effects of AMH level and age contributing to the prediction of POR were 95.3% and 1.8%, respectively. The incidences of POR increased with its predicted probability in both the model building and in external validation datasets, indicating its stability. An online website-based tool for assessing the score of ovarian reserve (http://121.43.113.123:9999) has been developed.

**Conclusions:**

Based on external validation data, the AA model performed well in predicting POR, and was more cost-effective and convenient than our previous published models.

## Introduction

Although the number and quality of ovarian follicles change profoundly with a woman’s age, the individual process of ovarian aging has not yet attracted enough attention (1). To our knowledge, most women do not know about the heterogeneity that is documented for ovarian reserve and ovarian aging. Irregular cycles and the menopause are easily identified, but fertility is already extremely low when these signs actually appear, leaving limited interventions available (2, 3). The decline of natural fertility occurs earlier, but often cannot be recognized directly (1).Almost 20% of reproductive-aged women attending infertility clinics show signs of premature ovarian aging. Such increased female infertility results from both the lack of knowledge on the heterogeneity of ovarian reserve/ovarian aging, and the tendency to delay childbearing in modern societies.

Ovarian reserve refers to the number of primordial follicles in the ovary that have the capacity to develop into mature oocytes (4–6). It is acknowledged that the age-related decrease in ovarian reserve is linked to the gradual loss of primordial follicles (1, 7), when follicular atresia takes place (8, 9).Anti-Müllerian hormone (AMH) is secreted by granulosa cells from preantral and small antral follicles (10, 11). One role of this hormone is to inhibit the recruitment of primordial follicles (12–14), so it is closely linked to ovarian reserve. The level of AMH reflects the number of gonadotropin (Gn)-independent small growing follicles (15), and the better the ovarian reserve is, the more such follicles there are.

We have already established two models for predicting ovarian reserve: a four-predictor AAFA (AMH–antral follicle count (AFC)–FSH–age) model (16), and a three-predictor AFA (AMH–FSH–age) model (17). The advantage of the AFA model is that on one hand, it does not need the AFC, only two serum hormone predictors and age, but the area under the curve (AUC) of the receiver operating curve (ROC) is as good as the AAFA model (17). However, blood sampling for FSH, the predictor in both models, needs to be done specifically on day 2 of the menstrual cycle, which limits the clinical application of the AFA model in general medical examinations. In this study, considering the known correlation between AMH and FSH levels, we aimed to establish an ‘AA model’ using only AMH and age, so that blood sampling could be carried out at any time in the menstrual cycle for assessing ovarian reserve.

## Materials and methods

### Subjects

This was a retrospective observational cohort study in Peking University Third Hospital. In all, 4796 standard gonadotropin releasing hormone (GnRH) antagonist cycles from 2017 to 2018 were selected according to inclusion and exclusion criteria for model building based on the datasets used in our previous studies (16, 17). Then, 5009 standard GnRH antagonist cycles from 2019 without sample selection were used for external validation. The inclusion criteria in the 2017-2018 data included: 1) female patients aged 20-45 y; 2) body mass index (BMI) ≤30 kg/m^2^; 3) previous attempted cycles ≤ 2. The exclusion criteria for the 2017-2018 data included: 1) treated or untreated ovarian cysts; 2) previous ovarian surgery; 3) polycystic ovary syndrome (PCOS); 4) previous metabolic or endocrinological diseases; 5) previous tuberculosis; 6) mild ovarian stimulation protocols; and 7) women with chromosomal abnormalities. The basic characteristics of the 2017-2018 model building data and the 2019 external validation data are shown in **Table 1**. The dataset used in this study contains deidentified data, so the need for informed consent from the patients was waived and institutional review board approval was not needed, thereby conforming to the Helsinki Declaration (18).

**Table 1:** Basic characteristics among women undergoing standard GnRH-antagonist cycles.

	selected 2017–2018 data (n=4796)	unselected 2019 data (n=5009)
Age (years)	32.9 ± 5.0	32.6 ± 4.7
BMI (kg/m^2^)	22.3 ± 2.9	22.8 ± 3.5
AMH (ng/ml)	2.5 (1.2-4.5)	2.5 (1.3-4.6)
basal FSH (IU/L)	6.8 (5.8-8.4)	6.7 (5.5-8.1)
AFC	10 (6-14)	10 (7-15)
NROs	10 (6-16)	11 (7-16)

If data fit normal distribution, Values represented as mean ± SD, if not, Value represented as median (quar); BMI, body mass index; AMH, Anti-Müllerian Hormone; FSH, Follicle Stimulating Hormone; AFC, antral follicle count; NROs, number of retrieved oocytes.

### Sampling and endocrine assays

Venous blood was collected using serum separation tubes that were inverted immediately and mixed five times, left at room temperature for 30 min, centrifuged at 1700 g for 10 min, and the serum retained for hormone measurements. The serum AMH level was measured on any day of the menstrual cycle, while the serum FSH level was measured on day 2. The assays and quality controls for FSH and AMH were as described (16, 17).

### Statistical analysis

Research data in this study were collected from the patient registry database in Peking University Third Hospital. All the analyses in this study were performed using SAS JMP Pro (version 14.2; SAS Institute, Cary, NC, USA), and *p* < 0.05 was considered statistically significant. Normally distributed variables are presented as the mean and standard deviation, while variables not normally distributed are presented as the median and quartiles. POR (<5 oocytes retrieved during ovarian stimulation) was used as the outcome variable for this AA model, which was the same as used previously for our AFA (17) and AAFA (16) models. Age and AMH were included into our modeling as categorical predictive variables. Least Absolute Shrinkage and Selection Operator (LASSO) logistic regression with 5-fold cross validation was applied to construct the model, and that with the lowest scaled log-likelihood was selected. The main effect of each predicting variable measures the variation over the distribution of *x*_j_, in the mean POR, which reflects the relative contribution of the variable alone to the model. Accuracy of the predictive AA model was assessed using the area under the ROC curve (AUC), sensitivity and specificity, with 95% confidence interval (CI) values. We also used the AUC and Venn diagrams to compare the accuracy of the AA model with the AFA and AAFA models (16, 17). Ranking of ovarian reserve from good to poor were based on the predicted probability of POR from low to high. Ovarian reserve, [score (N) = (1-predicted POR probability) × 100].

## Results

For building the AA model, first, the continuous variables of AMH and age were transformed into categorical variables, and the classification was mainly based on prior data exploration, as indicated in **Supplementary Figure 1**. To minimize the potential collinearity of variables measured from the same patient and over-fitting of variables, the LASSO logistic regression was used. In the 2017-2018 model-building dataset, we used five-fold cross-checking for internal validation. The AA model with the lowest scaled log-likelihood was finally selected. The AUCs for the training and internal validation sets were 0.862 and 0.843, respectively.

The effects of each predicting variable on the POR were listed in **Table 2**. Compared with the AMH control group (AMH ≤0.2 ng/ml), the parameter estimates decreased with AMH concentrations, indicating the decreasing probability of POR with increased AMH levels, which is of statistical significance. Compared with the age control group (≤ 30 y), the parameter estimates increased with age group, indicating the increasing probability of POR with increased age. The main effects of AMH and age contributing to this model were 95.3%, and 1.8% respectively. The actual incidences and predicted probabilities of POR in each group of the 60 groups in 2017-2018 data are listed in **Table 3**. This table can help us understand the performance of our model. Ideally, the predicted probability of each group should be exactly the same as the actual incidence.

**Table 2:** The effects of each predicting variable on POR using AA model.

Variables	Parameter estimation	Standard error	Wald χ^2^	*p* value
AMH [(3,~) vs [0,0.2]]	–4.335	0.234	343.3393	<.0001
AMH [(2,3] vs [0,0.2]]	–3.8031	0.252	227.7726	<.0001
AMH [(1.8,2] vs [0,0.2]]	–3.2347	0.3292	96.5493	<.0001
AMH [(1.4,1.8] vs [0,0.2]]	–2.883	0.245	138.4393	<.0001
AMH [(1.2,1.4] vs [0,0.2]]	–2.3813	0.2546	87.4919	<.0001
AMH [(1.0,1.2] vs [0,0.2]]	–2.2321	0.2458	82.4412	<.0001
AMH [(0.6,1.0] vs [0,0.2]]	–1.6751	0.2147	60.8473	<.0001
AMH [(0.4,0.6] vs [0,0.2]]	–0.8742	0.2357	13.7592	0.0002
AMH [(0.2,0.4] vs [0,0.2]]	–0.5727	0.2406	5.6686	0.0173
Age [(42,~) vs [0,30]]	0.807	0.2305	12.2579	0.0005
Age [(39,42] vs [0,30]]	1.0434	0.1711	37.1979	<.0001
Age [(37,39] vs [0,30]]	0.5885	0.1794	10.7573	0.001
Age [(35,37] vs [0,30]]	0.4299	0.1705	6.3561	0.0117
Age [(30,35] vs [0,30]]	0.28	0.14	4.06	0.04

**Table 3:** The actual incidence (predicted probability) of POR.

		Age classification (years)					
		≤30	(30, 35]	(35, 37]	(37, 39]	(39, 42]	>42
AMH classification (ng/ml)	(3,~]	2.3% (2.4%)	4.2% (3.2%)	0.8% (3.7%)	4.2% (4.3%)	4.6% (6.6%)	6.3% (5.3%)
	(2,3]	4.1% (4.1%)	5.6% (5.4%)	7.1% (6.2%)	6% (7.1%)	7.9% (10.8%)	25.0% (8.7%)
	(1.8,2]	13.3% (7.0%)	1.7% (9.1%)	9.4% (10.4%)	0% (11.9%)	25.0% (17.6%)	50.0% (14.4%)
	(1.4,1.8]	12.6% (9.7%)	11.687% (12.4%)	13.5% (14.1)	19.34% (16.2%)	16.4% (23.3%)	18.2% (19.3%)
	(1.2,1.4]	10.0% (15.0%)	20.3% (19.0%)	16.2% (21.4%)	16.7% (24.1%)	42.3% (33.4%)	33.3% (28.4%)
	(1.0,1.2]	9.% (17.0%)	20.0% (21.4%)	21.4% (24.0%)	40.0% (27.0%)	47.8% (36.8%)	10.0% (31.5%)
	(0.6,1.0]	30.4% (26.4%)	35.3% (32.2%)	33.9% (35.5%)	40.3% (39.2%)	37.5% (50.4%)	48.5% (44.5%)
	(0.4,0.6]	50.0% (44.4%)	44.4% (51.4%)	48.0% (55.1%)	54.6% (58.9%)	70.6% (69.4%)	72.7% (64.1%)
	(0.2,0.4]	54.6% (51.9%)	56.1% (58.9%)	73.7% (62.4%)	70.4% (66.0%)	81.0% (75.4%)	58.1% (70.7%)
	≤0.2	56.7% (65.5%)	75.0% (71.7%)	82.1% (84.6%)	76.9% (77.5%)	86.7% (84.4%)	79.2% (81.1%)

POR, poor ovarian response.

**Figure 1:**
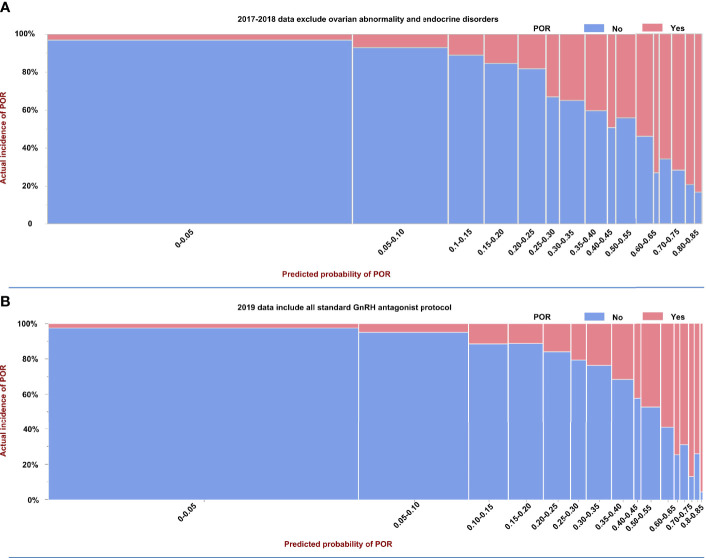
Prevalence and predicted probability of poor ovarian reserve (POR) in model building and external validation data. **(A)** Model building based on selected 2017–2018 data excluded women with ovarian abnormalities and endocrinopathies, and **(B)** the external validation data used unselected 2019 data including all standard GnRH antagonist protocols.

We collected additional 2019 data for external validation of this AA model. The difference from the 2017-2018 data is that these data did not undergo data selection, and all standard GnRH antagonist protocols were included. **Figure 1** shows the incidence of POR and predicted probability of POR in model-building data (2017-2018 data), which excluded women with ovarian abnormalities and endocrinopathies, and the external validation data (2019 data) including all standard GnRH antagonist protocols. **Figure 1** also contributes to the understanding of the performance of our model. Ideally, the predicted probability of each group should be exactly the same as the actual incidence. As shown, the prevalence of POR increased with its predicted probability in our AA model. For example, in women with the worst ovarian reserve, with a predicted POR probability of 0.8-0.85, the prevalence of POR was 20/21 in 2019 data vs 45/54 in the 2017-2018 data. The incidences and predicted probabilities of POR in 60 groups in the 2019 data are shown in **Supplementary Table 1**.

**Figure 2:**
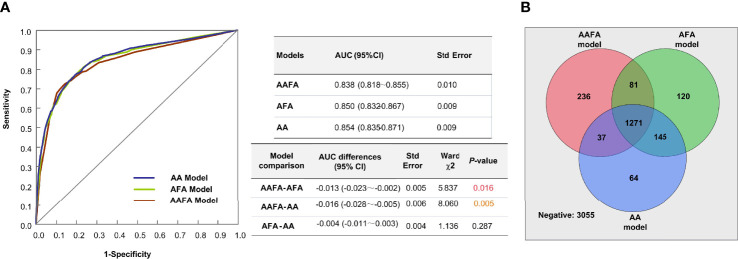
The performances of the three ovarian reserve models-AAFA, AFA, and AA-using the external validation dataset. **(A)** AUC Comparison of the three ovarian reserve models. For model comparison, AAFA-AFA means the AUC of AAFA minus the AUC of AFA, its result is indicated in the column of AUC differences. **(B)** The Venn diagram shows the predicted negative and positive POR estimates of the three models in this 2019 external verification data.

The performance of this AA ovarian reserve model we established here was compared with those of the AAFA (16), and AFA (17)models we built previously, using 2019 external validation data. **Figure 2A** demonstrates the AUCs and the differences between these three models. The AUCs of the AFA and AA models were significantly higher than in the AAFA model, while the difference between the AFA and AA model in the AUCs was not significant. The Venn diagram in **Figure 2B** shows the predicted negative and positive POR estimates of the three models in this 2019 external verification data. The numbers within circles indicate the predicted positive cases, while those outside the circles indicate the predicted negative cases. There were 1271 overlapping positive predicted cases of POR in the three models, and 3055 overlapping predicted negative cases of POR. The overlapping negative and positive predicted incidences in all three models accounted for 86.4% of the total 5009 samples in the 2019 data.

The above results showed that the AAFA model and the AFA model were not better than the AA model. We wondered whether it was caused by the different grouping standards of the independent variables in different models. Therefore, we explored the use of the same grouping criteria for AMH and age in the AA model and reassessed the AFA and AAFA models. The 2017-2018 data were used as a training set, and the 2019 data were used as aa validation set. The estimation parameters of the improved AAFA and AFA models are shown in **Supplementary Tables 2**, **3**, respectively. The performances of all three models in terms of the AUC, sensitivity, and specificity are shown in **Table 4**. No differences in term of AUC, sensitivity, and specificity were discovered, demonstrated by overlapping of their 95% confidence intervals. The ovarian reserve scores evaluated by AA model, improved AAFA and AFA models are almost the same, and the raw data and the ovarian reserve scores are shown in **Supplementary Table 4**.

**Table 4:** The AUCs, sensitivity and specificity of AA, improved AAFA and AFA models in training (2017-2018 data) and external validation (2019 data) using the same grouping criteria.

Measures	AA model		AAFA model		AFA model	
	training set	validation set	training set	validation set	training set	validation set
ROC (95% CI)	0.860 (0.850∼0.870)	0.854 (0.844 ∼ 0.864)	0.870 (0.858∼0.881)	0.882 (0.848∼0.908)	0.861 (0.848∼0.872)	0.875 (0.837∼0.905)
Sensitivity (95% CI)	0.485 (0.451∼0.520)	0.462 (0.423∼0.500)	0.463 (0.436∼0.490)	0.434 (0.357∼0.516)	0.412 (0.386∼0.439)	0.441 (0.363∼0.523)
Specificity (95% CI)	0.941 (0.933∼0.948)	0.961(0.955,0.966)	0.966 (0.962∼0.970)	0.958 (0.942∼0.969)	0.968 (0.963∼0.971)	0.959 (0.943∼0.971)

The contribution made by age is small, does it mean that AMH alone can be used to assess ovarian reserve? In addition, whether it is possible to use AMH as a continuous variable rather than transforming it into a categorical variable, we made the following comparison. As shown in **Table 5**, we constructed four models: model 1 with AMH + age as categorical variables; model 2 with AMH alone as a categorical variable; model 3 with AMH + age as continuous variables; model 4 with AMH alone as a continuous variable. As shown in **Table 5**, the AUCs of the four models were similar, but the sensitivities were lower in models 3 and 4 with large variations in sensitivity (i.e., the true positive rate) in the training and validation sets. Thus, AMH as a continuous variable was not optimal.

**Table 5:** Comparison of different models using AMH or not, or using AMH as categorical variable or not.

	Model-1 (AMH and age as categorical variables)		Model-2 (AMH as categorical variable)		Model-3 (AMH and age as continuous variables)		Model-4 (AMH as continuous variable)	
	Training	Validation	Training	Validation	Training	Validation	Training	Validation
AUC	0.86	0.85	0.85	0.85	0.86	0.86	0.86	0.86
Sensitivity	0.49	0.42	0.48	0.44	0.26	0.35	0.17	0.35
Specificity	0.94	0.97	0.95	0.96	0.98	0.98	0.99	0.98

Training, training set using 2017-2018 data; Validation, Validation set using 2019 data.

As to whether age is needed in ovarian reserve assessment. The aim of establishing this model is not only to predict POR or not (two groups), but also to rank the ovarian reserve of individuals into many subgroups according to the predicted probability of POR. AUC is only used to evaluate the performance of predicting POR or not, based on a predicted POR probability of 50% or not. In **Table 3**, we demonstrated the predicted probability and incidences of POR in all 60 groups in the 2019 external validation data. Obviously, the predicted POR probability and incidences of POR in different age groups within the same AMH group were significantly different. According to our ovarian reserve score, we ranked the ovarian reserve of individuals into many subgroups based on the predicted probability of POR, not just two groups of POR or not. Therefore, considering the clinical application, we chose to use both AMH and age as categorical variables for model building. **Table 3** showed that among the 60 groups, the predicted probabilities of POR were close to their incidences in each group. The same trend was also shown in **Figure 1**. These results indicated that our model 1 using AMH and age as categorical variables is of great practical value. Thus, we chose the model 1 as our final AA model.

**Figure 3:**
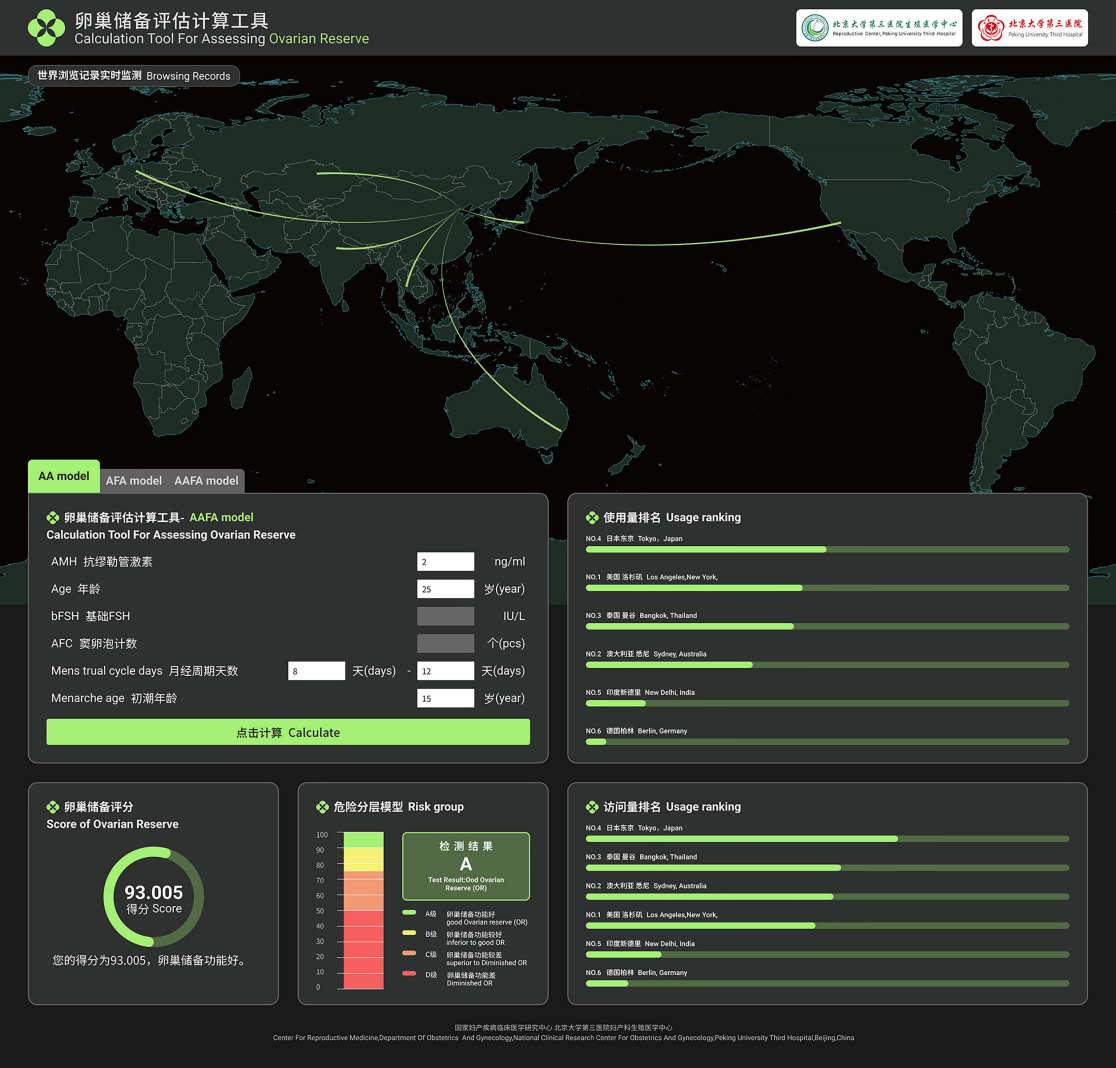
The website-based ovarian reserve assessment tool according to the AA model, improved AAFA model and improved AFA model.

Next, we constructed a website-based ovarian reserve assessment tool according to the AA model, and the improved AAFA and AFA models (http://121.43.113.123:9999) (**Figure 3**). The input information also includes age at menarche, menstrual cycle duration and other related basic information, which are designed for further improvement of this software. The result display will give the user an estimate of their ovarian reserve, up to a full score of 100 points. The higher the score, the better the predicted ovarian reserve [score (N) = (1–predicted POR probability) ×100]. The grouping criteria of ovarian reserve from A to D are consistent with our previous studies (16, 17).

## Discussion

Here we have established an optimized two-indicator AA model for assessing ovarian reserve, which has the advantage of being applicable at any time during the menstrual cycles compared with our previous AFA (17) and AAFA (16) models. Moreover, with fewer indicators, the cost is lower and the performance of the AA model is similar to the improved AFA and AAFA models. In the same 2019 external validation dataset using the same grouping criteria, the AUCs of AA, improved AAFA and improved AFA models were 0.854 (0.844-0.864), 0.882 (0.848-0.908), and 0.875 (0.837–0.905), indicating that there are no significant differences among these models.

Assisted reproductive technology (ART) approaches are often ineffective in women with diminished ovarian reserve (DOR) (2, 3), thus increasing the economic, medical and social costs of ART. The advent of our online tool will be of great help for reproductive-aged women to arrange their childbearing plans according to their own ovarian reserve status, and might help in reducing the incidence of infertility by giving a timely warning of DOR. Besides, our online ovarian reserve assessment tool could not only help a reproductive woman know about her ovarian reserve status but might also be applied to infertility related medical research. For example, the role of the DNA fragmentation index (DFI) on male fertility is inconclusive (19–21), because of the compounding factors such as DOR. Using our online tool for estimating ovarian reserve, this acknowledged factor for female fertility will be controlled, so the role of DFI in the fertility of couples will be clarified.

How to transform the continuous variable of AMH level into a categorical variable according to cut-offs was a critical decision for our analysis. First, it is mainly based on data exploration, that is, to explore the best grouping method according to the relationship between independent variables and the outcome variable. Taking the AMH level as an example, we initially divided it into many groups with an interval of 0.1ng/ml, and then pooled groups with similar incidences of POR. Second, to pool those groups, in addition to the data exploration, the clinical significance or clinical experience needs to be considered. Third, it is very important to ensure that there are enough cases in each category to avoid unstable results. Considering the above three points, we finally divided the AMH level into 10 categories.

The dynamic changes in AMH levels and in the numbers of primordial follicles (i.e., ovarian reserve) with age were subjects in our recent reviews (22–25). The number of primordial follicles drops rapidly before the initial expression of AMH in the fetus, at 36 gestational weeks (11), and then slows down after the advent of AMH secretion. After menarche, both the numbers of primordial follicles and the AMH level decrease with age. Therefore, AMH has increasingly been regarded as the best marker for ovarian reserve in recent years. The main effect of AMH in our AA model is 95.3%, and age contributes to only 1.8%, indicating the key role of AMH in assessing ovarian reserve.

Why do we define POR using the number of oocytes retrieved rather than the number of MII oocytes and 2PN fertilized embryos as the outcome variable for ovarian reserve assessment? Our results indicate that AMH and age as categorical variables can also predict the number of MII oocytes and 2PN fertilized embryos, but with lower AUC and lower sensitivity (i.e., the true positive rate) (**Supplementary Table 5**). This might because the AMH level and age mainly reflect the basic status of ovarian reserve, but when ART progresses, the numbers of MII oocytes and 2PN fertilized embryos are not only affected by ovarian reserve, but also other factors, for example, the FSH receptor status and male factors. Therefore, we suppose that the outcome variable of POR defined by the number of oocytes retrieved might be a better outcome variable for ranking ovarian reserve among reproductive-age women. Other variables such as the number of aspirated follicles on the day of oocyte retrieval might be a good candidate for outcome variable, which needs further investigation.

As to the effectiveness with extreme values of AMH, one of the reasons we chose to build this model using AMH and age as categorical variables is its stability, as described above. Furthermore, we have also developed a PCOS screening model (26) (http://121.43.113.123:8888/). A woman with an extremely high AMH level could first be screened for PCOS, and then be assessed for ovarian reserve status using our models.

### Limitations of the study

As to the limitations of our study, first, because the AA model is divided into 60 subgroups, we found that the predicted probability of POR in some groups differed from the actual incidence of POR as listed in **Table 4**. This might reflect the small sample sizes in certain subgroups, but through expanding sample sizes and re-classification of the predictors, we believe that the performances of the AA, AFA and AAFA models will be improved in the future. Second, representativeness was an issue. Our original hypothesis was that the order of ovarian reserve from good to poor in patients undergoing GnRH antagonist ovarian stimulation would reflect the order of ovarian reserve from good to poor in the general population; however, this assumption needs to be tested. However, ovarian reserve can not be tested noninvasively, posing ethical challenges for further verification of our AA ovarian reserve model in the general population.

## Data availability statement

The original contributions presented in the study are included in the article/**supplementary material**. Further inquiries can be directed to the corresponding author.

## Ethics statement

The studies involving human participants were reviewed and approved by The Human Reproductive Ethics Committee of Peking University Third Hospital. The patients/participants provided their written informed consent to participate in this study.

## Author contributions

YH, HX and GF contributed to manuscript drafting and revising. YH, HX, GF, HW, LC, and KA contributed to data analysis and interpretation. HX, HW, LC, and MZ prepared figures and tables. RL contributed to the conception of the study, manuscript revising and final approval. All authors reviewed the manuscript.

## Funding

This study was supported by the National Key Research and Development Program of China (Grant No. 2018YFC1002104, 2018YFC1002106); the Innovation & Transfer Fund of Peking University Third Hospital (Grant No. BYSYZHZB2020102, BYSYZHKC2021104); the National Natural Science Foundation of China for Distinguished Young Scholars (Grant No. 81925013); the Major National R&D Projects of China (Grant No. 2017ZX09304012-012); National Natural Science Foundation of China (Grant No. 81771650); the Capital Health Research and Development of Special Project (Grant No. 2018-1-4091).

## Conflict of interest

Author KA is employed by The Predict Health.

The remaining authors declare that the research was conducted in the absence of any commercial or financial relationships that could be construed as a potential conflict of interest.

## Publisher’s note

All claims expressed in this article are solely those of the authors and do not necessarily represent those of their affiliated organizations, or those of the publisher, the editors and the reviewers. Any product that may be evaluated in this article, or claim that may be made by its manufacturer, is not guaranteed or endorsed by the publisher.
