# Supplementary material for: An online tool for predicting ovarian reserve based on AMH level and age: A retrospective cohort study
Source: Front Endocrinol (Lausanne). 2022 Jul 22;13:946123. doi: 10.3389/fendo.2022.946123 (PMC9353219; doi:10.3389/fendo.2022.946123)
Supplement: Supplementary file 1 [file DataSheet_1.docx]

**Correlation coefficient (r)**

|  | **age** | **LN [FSH]** | **LN[AMH]** | **LN[AFC]** |
| --- | --- | --- | --- | --- |
| age | 1.0000 | 0.1951 | -0.4175 | -0.4260 |
| log[FSH] | 0.1951 | 1.0000 | -0.3018 | -0.2867 |
| log[AMH] | -0.4175 | -0.3018 | 1.0000 | 0.6769 |
| log[AFC] | -0.4260 | -0.2867 | 0.6769 | 1.0000 |

**Confidential interval (CI) of r**

| **variables** | **variables** | **r** | **Lower limit of 95% CI** | **Upper limit of 95% CI** |
| --- | --- | --- | --- | --- |
| ln [FSH] | age | 0.1951 | 0.1678 | 0.2222 |
| ln[AMH] | age | -0.4175 | -0.4406 | -0.3939 |
| ln[AMH] | ln[FSH] | -0.3018 | -0.3272 | -0.2758 |
| ln[AFC] | age | -0.4260 | -0.4488 | -0.4026 |
| ln[AFC] | ln[FSH] | -0.2867 | -0.3125 | -0.2606 |
| ln[AFC] | ln[AMH] | 0.6769 | 0.6613 | 0.6920 |

**Matrix of Scatter Plot**
