# Supplementary figures and images for: An online tool for predicting ovarian reserve based on AMH level and age: A retrospective cohort study
Source: Front Endocrinol (Lausanne). 2022 Jul 22;13:946123. doi: 10.3389/fendo.2022.946123 (PMC9353219; doi:10.3389/fendo.2022.946123)

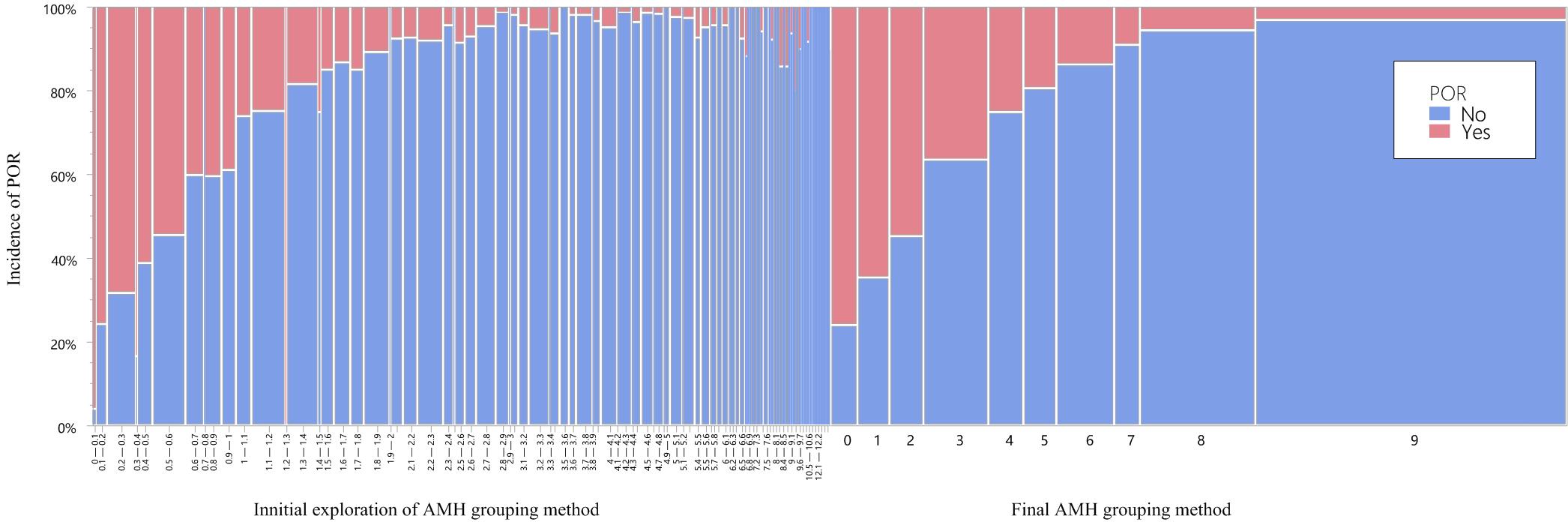

Supplement: Supplementary file 2 [file Image_1.jpeg]
